# Age-dependent increase in peripheral neuropeptide Y with no cross-sectional link to depression

**DOI:** 10.1017/neu.2026.10084

**Published:** 2026-05-08

**Authors:** Mehdi Dahlaki, M.D. Zayeed Alam, Marit Nyholm Nielsen, Henriette Nørmølle Buttenschon, Michael Winterdahl

**Affiliations:** 1 Translational Neuropsychiatry Unit, Aarhus University, Denmark; 2 Centre for Research and Education, Gødstrup Hospital, Denmark; 3 Department of Clinical Medicine, Aarhus University, Denmark; 4 Aalborg University Hospital, Denmark

**Keywords:** neuropeptide Y, depression, stress resilience, ageing, plasma biomarkers

## Abstract

**Background:**

Neuropeptide Y (NPY) has been implicated in stress resilience and depression, yet findings on circulating levels in major depressive disorder (MDD) remain inconsistent, possibly due to confounders such as age, sex, and body mass index (BMI). This study assessed NPY concentrations in individuals with MDD and in matched controls from the Danish PRISME cohort of public-sector employees.

**Methods:**

We investigated plasma NPY (p-NPY) concentrations in 77 adults with a current ICD-10 diagnosis of MDD and 77 age- and sex-matched controls. Plasma samples were analysed using a commercial ELISA kit. A general linear model examined the effects of group, age, sex, and BMI, including a sex-by-group interaction. Plasma NPY values were log_10_-transformed prior to analysis.

**Results:**

p-NPY concentrations did not differ significantly between MDD and controls (*p* = 0.785). No main effect of sex or sex-by-group interaction was observed (all *p* > 0.17), and BMI was unrelated to p-NPY (*p* = 0.917). By contrast, age was a strong predictor (β = 0.38, *p* < 0.001), explaining 13% of the variance.

**Conclusions:**

In this community-based sample of high-functioning individuals with MDD, we found no evidence of altered p-NPY concentrations compared with matched controls. Instead, age emerged as a robust determinant of circulating NPY levels, independent of depression status and BMI, after adjustment for sex. These findings suggest that peripheral NPY variation may be more strongly related to demographic factors than to depression per se in this type of cohort.

Significant outcomes

- Plasma neuropeptide Y (p-NPY) levels did not differ between high-functioning adults with major depressive disorder and matched healthy controls in this cohort.
- Age was strongly associated with higher p-NPY concentrations, independent of depression status, sex, and BMI.
- Demographic factors, especially age, must be considered when interpreting peripheral neuropeptide biomarkers in both clinical and non-clinical populations.

Limitations

- The cross-sectional design precludes causal inference and may overlook state-dependent fluctuations in NPY.
- Limited clinical characterisation (e.g. symptom severity, illness duration, treatment resistance) limits the interpretation of illness-stage effects.
- The cohort consists of employed high-functioning individuals, limiting the generalisability to more severe or treatment-resistant depression.
- Unequal sex distribution (predominantly women) reduced the power to detect sex-specific effects.

## Introduction

Major depressive disorder (MDD) is a leading cause of disability worldwide, characterised by persistent low mood or loss of interest in previously rewarding activities. Depressive disorders rank as the third leading cause of non-fatal health loss in women and the fifth in men, and the associated years lived with disability have increased steadily over recent decades (James *et al*., 2018).

Neuropeptide Y (NPY), one of the most abundant neuropeptides in the mammalian brain, has attracted considerable interest as a modulator of stress resilience and emotional regulation. Experimental evidence supports a role for NPY in affective regulation: pharmacological and neuromodulatory interventions increase central NPY signalling and exert antidepressant-like effects in rodents (Heilig *et al*., 1988; Madsen *et al*., 2000), while non-pharmacological interventions such as mindfulness training have been associated with increased peripheral NPY concentrations in stressed individuals (Østergaard *et al*., 2025). Peripheral NPY has also been linked to psychosocial traits in healthy populations, including religious commitment (Tønnesen *et al*., 2019). Beyond emotional regulation, NPY is a key regulator of energy homeostasis and feeding behaviour; central administration induces hyperphagia and weight gain in rodents (Glenn Stanley *et al*., 1986), whereas diet-induced obesity is associated with reduced hypothalamic NPY expression (Hansen *et al*., 2004).

A recent meta-analysis reported lower circulating plasma NPY levels in patients with MDD compared with healthy controls, while no significant group differences were observed in cerebrospinal fluid (CSF) concentrations (Tural and Iosifescu 2020). Importantly, however, this summary effect may mask substantial heterogeneity across primary studies, which may reflect the non-linear regulation of NPY in response to stress. NPY has been described as a physiological buffer against stress, showing compensatory increases during acute or moderate stress exposure, and reductions only under chronic or severe conditions (Morgan *et al*., 2002; Heilig 2004; Reichmann and Holzer 2016). This non-linear, state-dependent profile suggests that some of the observed variation may be attributable to differences in the populations studied.

Depression itself is influenced by several biological factors, notably sex, age, and body mass index (BMI), though the direction and magnitude of these associations differ across populations. For example, a nationally representative Korean study reported a reduced risk of depression among overweight and obese older adults (≥65 years) of both sexes, with no corresponding pattern in younger age groups (Oh *et al*., 2018). Conversely, other work has identified positive associations between obesity, particularly visceral adiposity, and depression in women but not in men (Li *et al*., 2017).

Age, sex, and BMI also influence circulating NPY concentrations. In healthy populations, age-related trajectories appear sex-specific: NPY concentrations increase with age in women (Taniguchi *et al*., 1994; El Khoury and Mathé 2004), whereas male animals show declining hypothalamic NPY secretion with ageing (Sahu *et al*., 1988, 1990). Reviews further highlight pronounced sex differences in NPY expression and receptor sensitivity, with potential relevance for stress-related psychopathology (Nahvi *et al*., 2020). Findings in obesity are similarly inconsistent. Reduced serum NPY has been reported in overweight or obese adolescents (Saranac *et al*., 2007; Tyszkiewicz-Nwafor *et al*., 2021), whereas studies in adults have observed higher (Baranowska *et al*., 2005; Baltazi *et al*., 2011) or unchanged plasma concentrations (Milewicz *et al*., 2000; Nam *et al*., 2001). Taken together, these observations suggest that demographic and metabolic factors may substantially confound reported associations between peripheral NPY and depression, thereby contributing to the heterogeneity of the existing literature.

The present study was designed not as a biomarker discovery effort, nor as a simple replication of prior findings, but as a methodologically controlled evaluation of peripheral NPY in a well-characterised, high-functioning population. Using strict age matching and simultaneous adjustment for sex and BMI, we examined plasma NPY (p-NPY) concentrations in individuals with MDD and matched controls, with particular emphasis on age-related variance. The primary aim was to assess the robustness of previously reported peripheral NPY differences under tightly controlled demographic conditions.

## Methods

### Study population

Participants were drawn from the Danish PRISME study (Psychological RISk factors in the working environment and biological MEchanism for the development of stress, burn-out, and depression), a large prospective cohort of municipal and hospital employees in Aarhus County, Denmark (Kaerlev *et al*., 2011; Kolstad *et al*., 2011).

The present analysis included 77 individuals diagnosed with a current depressive episode according to ICD-10 criteria and 77 age- and sex-matched controls with no history of affective or anxiety disorders. Diagnostic status was established using the Schedules for Clinical Assessment in Neuropsychiatry (SCAN, version 2.1), administered by trained clinicians (Wing *et al*., 1990). Demographic data, including age and sex, were obtained via linkage with the Danish Civil Registration System (Pedersen *et al*., 2006). BMI was calculated as weight in kilograms divided by height in metres squared (kg/m^2^), based on height and weight measured during the first clinical assessment by trained clinical staff using standardised procedures. Measurements were obtained under non-fasted conditions. Table 1 summarises the characteristics of the MDD and control groups, which were comparable in age, sex distribution, and BMI.

**Table 1.** Characteristics of participants

Characteristic	Control (*N* = 77)	Depressed (*N* = 77)
**Sex**		
Women	66	66
Men	11	11
**Age (years)**		
Mean (SD)	45.51 (8.33)	45.53 (9.27)
**BMI (kg/m^2^)**		
Mean (SD)	24.96 (4.17)	26.35 (6.06)

Demographic and anthropometric characteristics of depressed and control participants. Values are mean (SD).

All participants provided written informed consent prior to enrolment. The study was approved by the Danish Data Protection Agency (2006-41-7032) and the Danish Regional Ethics Committee (RRS 2006-1028/2747-06).

### Biological measures

The primary outcome was plasma neuropeptide Y (p-NPY) concentration. Blood samples were collected in 2007, stored continuously at −80°C, and analysed in 2020 at the Translational Neuropsychiatry Unit, Aarhus University. All samples were stored under identical conditions and were not subjected to repeated freeze–thaw cycles prior to analysis. Samples were assayed within the same analytical period, storage duration did not differ between groups, and all samples were processed in parallel to minimise potential batch effects.

Concentrations were quantified using a commercially available Human NPY Sandwich ELISA Kit (96-well strip plate, Cat. #EZHNPY-25K, Sigma-Aldrich/Merck), following the manufacturer’s protocol.

NPY concentrations were calculated by interpolation from standard curves generated on each plate using a four-parameter logistic fit, and duplicate measurements were averaged for analysis. All seven assay plates were derived from the same ELISA kit batch and were processed within a single run. Samples were randomised across assay plates to minimise systematic error. Intra-assay coefficients of variation, calculated from duplicate measurements, were approximately 1–2%. Inter-assay variability, assessed across plates processed using the same kit batch and calibration standards, was below 8%. Plate effects were assessed and found to be non-significant.

### Statistical analysis

All analyses were conducted in *Jamovi* (version 2.6; The Jamovi Project, 2024). P-NPY concentrations were log_10_-transformed to reduce skewness. A general linear model (GLM) tested the association between depression status and p-NPY, adjusting for age, sex, and BMI, and including a sex-by-group interaction.

We report β coefficients with 95% confidence intervals (CIs) for fixed effects, as well as partial eta squared (η^2^ *_p_*) from omnibus tests as measures of effect size. Statistical significance was set at *p* < 0.05 (two-tailed). Assumptions of normality and homogeneity of variance were evaluated using Shapiro–Wilk and Kolmogorov–Smirnov tests, Levene’s test, and inspection of residual plots (histograms, Q–Q plots, residual vs. predicted scatterplots).

To assess potential non-linear associations between age and p-NPY, an additional model including a quadratic age term was tested.

## Results

The overall model predicting log_10_-transformed p-NPY was statistically significant, *F*(5, 147) = 5.63, *p* < 0.001, explaining 16% of the variance (adjusted *R* ^2^ = 0.13; Table 2).

**Table 2.** General linear model of log_10_-transformed plasma NPY concentrations with predictors age, sex, BMI, group, and interaction Table 2 long description.

Predictor	Estimate	SE	95% CI [LL, UL]	B	DF	T	*P*
Intercept	1.46	0.02	[1.43, 1.50]	–	147	83.15	<0.001
Age	0.01	0.00	[0.00, 0.01]	0.38	147	4.95	<0.001
BMI	0.00	0.00	[−0.00, 0.00]	0.01	147	0.10	0.917
Sex (male vs. female)	0.05	0.04	[−0.02, 0.12]	0.29	147	1.37	0.174
Group (MDD vs. control)	−0.01	0.04	[−0.08, 0.06]	−0.06	147	−0.27	0.785
Sex × Group	−0.02	0.07	[−0.16, 0.12]	−0.11	147	−0.26	0.795

General linear model predicting log_10_-transformed plasma NPY concentrations. Estimates are unstandardised regression coefficients with standard errors (SE), 95% confidence intervals (CI), standardised coefficients (β), degrees of freedom (df), *t*-statistics, and *p*-values. Group coded as MDD = 1, control = 0; sex coded as male = 1, female = 0.

Contrary to our hypothesis, depression status did not predict p-NPY concentrations, *F*(1, 147) = 0.07, *p* = 0.785, η^2^ *_p_* < 0.001. Neither sex nor the sex × group interaction was statistically significant (all *p* > 0.17); however, these analyses were underpowered due to the small number of male participants and should be considered exploratory.

**Figure 1.**
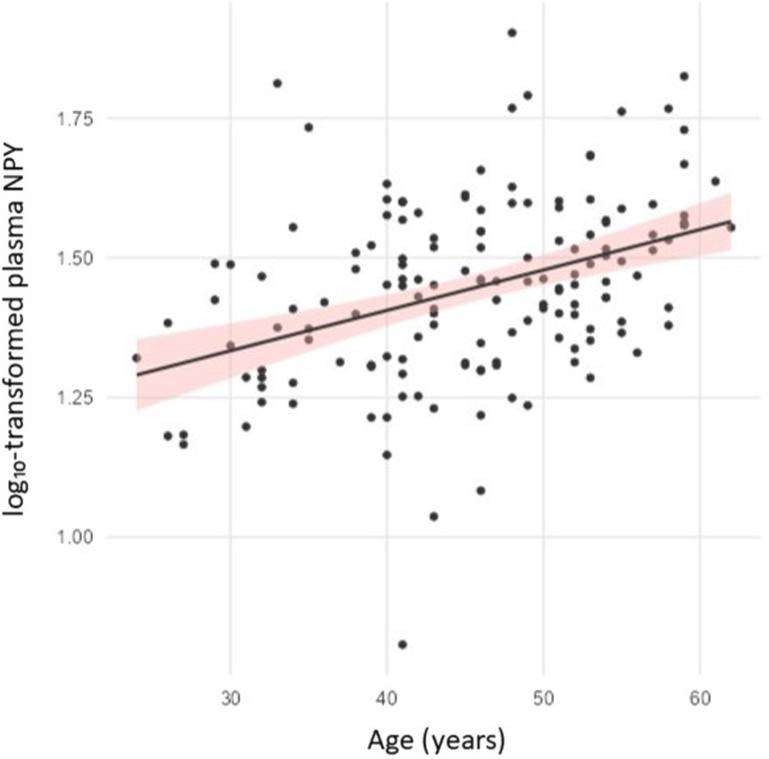
Figure 1 long description. Association between age and log_10_-transformed plasma NPY concentrations. Scatter plot showing the relationship between age and log_10_-transformed plasma NPY (p-NPY) concentrations and Age. Each point represents one participant. Regression lines indicate a positive association between age and p-NPY, with no significant difference in slope or intercept between groups.

By contrast, age emerged as a robust predictor of p-NPY, *F*(1, 147) = 24.47, *p* < 0.001, η^2^ *_p_* = 0.14. Higher age was associated with higher p-NPY concentrations (β = 0.38, 95% CI [0.00, 0.01]), a relationship illustrated in Fig. 1. On the log_10_ scale, this corresponds approximately to a 2–3% increase in p-NPY per decade of age. Inclusion of a quadratic age term did not improve model fit, and the non-linear term was non-significant (*p* = 0.325), supporting a linear association between age and p-NPY. No significant age × group interaction was observed, indicating comparable age-related slopes across groups.

Model assumptions were generally met. Levene’s test indicated homogeneity of variance (*F*(3, 149) = 0.58, *p* = 0.626). Residuals were approximately normally distributed on Q–Q plots, although Shapiro–Wilk testing indicated mild deviation (*p* = 0.011). Given the sample size, the GLM is considered robust to this violation.

## Discussion

In this population-based sample of high-functioning individuals, we found no evidence for altered p-NPY concentrations in individuals with MDD compared with matched controls. Instead, age emerged as the dominant explanatory factor, with higher circulating p-NPY concentrations observed in older participants, independent of depression status, sex, or BMI.

This absence of case–control differences contrasts with several prior reports and highlights the marked heterogeneity in the literature on peripheral NPY in depression. Across studies, plasma and serum measures have yielded lower (Hashimoto *et al*., 1996; Nilsson *et al*., 1996; Westrin *et al*., 1999; Gulec *et al*., 2010; Ozsoy *et al*., 2016; Xie *et al*., 2018), higher (Cizza *et al*., 2008), or unchanged (Irwin *et al*., 1991; Czermak *et al*., 2008; Nishi *et al*., 2014; Zheng *et al*., 2016) NPY concentrations in depressed cohorts relative to controls. Such inconsistency has complicated the interpretation of peripheral NPY as a biomarker of depression.

Cohort characteristics may help explain the absence of group differences. Unlike most clinical case–-control studies, the PRISME sample was drawn from employed municipal and hospital staff, predominantly women of medium to high social class, with relatively low rates of long-term sick leave (Kaerlev *et al*., 2011; Kolstad *et al*., 2011). As such, the present findings should be interpreted as reflecting a relatively resilient subgroup of individuals with MDD, in whom biological differences may be attenuated. In line with this interpretation, physical activity has been associated with elevated NPY concentrations in physically active individuals, even when baseline levels do not differ from those of controls (Zajadacz *et al*., 2009). Such functional resilience may have attenuated NPY differences between depressed and non-depressed participants in the present study.

It is also plausible that NPY alterations are state-dependent rather than trait-like. Changes in peripheral NPY may emerge primarily in response to acute stress, when the peptide is mobilised as part of the physiological stress-buffering system. Reduced NPY concentrations may also characterise patients with chronic, severe, or treatment-resistant depression. Although such cases may have been present in our cohort, the absence of detailed measures of illness duration, severity, or treatment resistance prevents us from determining their contribution to the present findings. Supporting a state-dependent interpretation, our group and others have previously linked lower plasma NPY concentrations to stress-related psychiatric conditions, including psychogenic non-epileptic seizures (Winterdahl *et al*., 2017; Miani *et al*., 2019).

One additional likely contributor to this heterogeneity observed in the literature is insufficient control for demographic and metabolic covariates known to influence circulating NPY. Many prior investigations did not adequately account for factors such as age, sex, or BMI, which could potentially influence peripheral neuropeptide levels. By adjusting for these variables, we demonstrate that age explains a meaningful proportion of variance in p-NPY concentrations and may obscure group-level differences when left uncontrolled. The strong positive association between age and p-NPY concentrations aligns with previous findings in healthy populations. Prior studies have reported increasing NPY levels with advancing age in women (Taniguchi *et al*., 1994; El Khoury and Mathé 2004), whereas animal studies demonstrate declining hypothalamic NPY secretion in ageing males (Sahu *et al*., 1988, 1990). These apparently sex-specific trajectories may reflect age-related changes in hypothalamic function, neurovascular integrity, or peripheral stress-regulatory pathways, although the underlying mechanisms remain incompletely understood. Our findings underscore that age-related variation in NPY is substantial and must be carefully considered in future biomarker research on depression.

While preclinical studies consistently report sex differences in NPY expression and receptor sensitivity (Nahvi 2020), findings in human plasma are mixed. Hormonal status, including menopausal stage and circulating sex steroid levels, may be more relevant than sex per se in shaping peripheral NPY concentrations, particularly in female-dominated samples. In the present study, the markedly unbalanced sex distribution (66 women vs. 11 men per group) reduced statistical power to detect sex-specific effects.

Similarly, BMI was not associated with p-NPY concentrations, despite prior studies reporting higher (Baranowska *et al*., 2005; Baltazi *et al*., 2011), lower (Saranac *et al*., 2007; Tyszkiewicz-Nwafor *et al*., 2021), or unchanged levels in obesity (Milewicz *et al*., 2000; Nam *et al*., 2001). These inconsistencies likely reflect differences in age distributions, hormonal status, and metabolic phenotypes across cohorts. In addition, other potential modulators of NPY, including physical activity, smoking status, hormonal status, medication use, and acute stress at sampling, were not available and may represent residual sources of confounding.

With 77 participants per group, the study had approximately 80% power to detect medium-sized effects (Cohen’s *d* ≈ 0.46) in p-NPY concentrations between patients and controls. Smaller effects (*d* ≤ 0.30) may therefore have gone undetected. However, the clinical relevance of such subtle differences is questionable, particularly in the context of biomarker development, where effect sizes must be sufficiently robust to support diagnostic or prognostic utility. The absence of medium- or large-group differences in this sample suggests that peripheral NPY is unlikely to serve as a broadly applicable clinical biomarker for MDD, although smaller effects or those confined to specific clinical subgroups cannot be excluded.

Several limitations should be acknowledged. First, the cross-sectional design precludes causal inference and may have overlooked transient or state-dependent changes in NPY. Second, although all patients were diagnosed according to ICD-10 criteria, continuous measures of depression severity and illness duration were not available, limiting symptom-level analyses. Third, plasma NPY represents an accessible but indirect proxy of central neuropeptide signalling, which can be assessed more directly using receptor-specific PET imaging approaches (Winterdahl *et al*., 2014). Finally, blood samples were collected in 2007 and analysed in 2020. While long-term storage at −80 °C is standard practice in biobanking (Peakman & Elliott, 2010; Sudlow *et al*., 2015) and generally preserves peptide integrity, extended storage may influence absolute concentrations. However, all samples were stored under identical conditions for comparable durations, were not subjected to repeated freeze–thaw cycles, and were analysed within the same batch. Any potential storage-related degradation would therefore be expected to affect all samples similarly and is unlikely to have introduced systematic bias between groups.

In this population-based sample of high-functioning, occupationally active individuals, we found no evidence of altered p-NPY concentrations in individuals with MDD compared with matched controls. Instead, age emerged as a robust determinant of circulating NPY levels, independent of depression status and BMI. These findings suggest that variation in peripheral NPY may be more strongly related to demographic factors than to depression per se in this type of cohort. However, the present results should be interpreted within the context of a relatively resilient and restricted phenotype of MDD and may not generalise to more severe, chronic, or treatment-resistant populations. Future studies should examine NPY dynamics in clinically diverse samples and under conditions of acute stress to further clarify its role in depression.

## Table 2. Long description

A table presents a general linear model predicting log10-transformed plasma NPY concentrations. The table includes predictors such as Intercept, Age, BMI, Sex, Group, and Sex x Group, each with corresponding estimates, standard errors, 95% confidence intervals, standardised coefficients, degrees of freedom, t-statistics, and p-values. The Intercept has an estimate of 1.46 with a p-value of less than 0.001. Age shows a positive estimate of 0.01 with a p-value of less than 0.001. BMI has an estimate of 0.00 with a p-value of 0.917. Sex (male vs. female) has an estimate of 0.05 with a p-value of 0.174. Group (MDD vs. control) has an estimate of -0.01 with a p-value of 0.785. The interaction between Sex and Group has an estimate of -0.02 with a p-value of 0.795.

Navigate back to Table 2..

## Figure 1. Long description

A scatter plot with dozens of data points showing the relationship between age and log10-transformed plasma NPY concentrations. The x-axis represents age in years, ranging from 30 to 60. The y-axis represents log10-transformed plasma NPY concentrations, ranging from 1.00 to 1.75. Each black dot represents one participant. A black regression line with a red shaded confidence interval indicates a positive association between age and plasma NPY concentrations. The data points are scattered with no significant clusters or outliers. All values are approximated.

Navigate back to Figure 1..
